# The P2X4 receptor is required for neuroprotection via ischemic preconditioning

**DOI:** 10.1038/srep25893

**Published:** 2016-05-13

**Authors:** Tomohiko Ozaki, Rieko Muramatsu, Miwa Sasai, Masahiro Yamamoto, Yoshiaki Kubota, Toshiyuki Fujinaka, Toshiki Yoshimine, Toshihide Yamashita

**Affiliations:** 1 Department of Molecular Neuroscience, Graduate School of Medicine, Osaka University, 2-2 Yamada-oka, Suita, Osaka 565-0871, Japan; 2 Core Research for Evolutional Science and Technology, Japan Science and Technology Agency, 5, Sanbancho, Chiyoda-ku, Tokyo 102-0075, Japan; 3 Department of Neurosurgery, Graduate School of Medicine, Osaka University, 2-2 Yamada-oka, Suita, Osaka 565-0871, Japan; 4 Precursory Research for Embryonic Science and Technology, Japan Science and Technology Agency, 5, Sanbancho, Chiyoda-ku, Tokyo 102-0075, Japan; 5 Department of Immunoparasitology, Research Institute for Microbial Diseases, Osaka University, 3-1 Yamada-oka, Suita, Osaka 565-0871, Japan; 6 The Laboratory of Vascular Biology, School of Medicine, Keio University, 35 Shinanomachi, Shinjuku-ku, Tokyo 160-8582, Japan

## Abstract

Ischemic preconditioning (IPC), a procedure consisting of transient ischemia and subsequent reperfusion, provides ischemic tolerance against prolonged ischemia in the brain. Although the blood flow changes mediated by IPC are primarily perceived by vascular endothelial cells, the role of these cells in ischemic tolerance has not been fully clarified. In this study, we found that the P2X4 receptor, which is abundantly expressed in vascular endothelial cells, is required for ischemic tolerance following middle artery occlusion (MCAO) in mice. Mechanistically, the P2X4 receptor was stimulated by fluid shear stress, which mimics reperfusion, thus promoting the increased expression of osteopontin, a neuroprotective molecule. Furthermore, we found that the intracerebroventricular administration of osteopontin was sufficient to exert a neuroprotective effect mediated by preconditioning-stimulated P2X4 receptor activation. These results demonstrate a novel mechanism whereby vascular endothelial cells are involved in ischemic tolerance.

Ischemic brain injuries resulting from decreases in cerebral blood flow (CBF) are important causes of disability and death. Pharmacological interventions are either ineffective or confounded by adverse effects, and neuroprotection in patients after ischemic brain damage consequently remains a major unfulfilled medical need. Ischemic preconditioning (IPC), defined as transient ischemia and subsequent reperfusion, exerts neuroprotective effects against lethal ischemia. Ischemic tolerance due to preconditioning is observed in experimental animal models. In humans, the number of investigations is currently limited, but ischemic tolerance may naturally occur in human patients^1^. Therefore, the mechanisms of IPC have attracted considerable attention in terms of their potential applications in adaptive medicine for the treatment of ischemia. Regarding the mechanisms underlying the acquisition of ischemic tolerance, most research has focused on the involvement of neurons and astrocytes. For example, the N-methyl-D-aspartate receptor and the adenosine A1 receptor, both of which are abundantly expressed in neurons^2^,^3^, are involved in preconditioning-induced ischemic tolerance^4^,^5^. The inhibition of astrocyte-expressed P2X7 receptors attenuates the induction of ischemic tolerance^6^. In contrast, although IPC is caused by transient blood flow changes^1^ that are directly perceived by vascular endothelial cells^7^, the role of these cells in ischemic tolerance has not been clarified.

Vascular endothelial cells are exposed to shear stress, in the form of the frictional force exerted by flowing blood. Shear stress mediates the molecular expression in vascular endothelial cells^8^ and influences vascular phenomena, such as angiogenesis^9^, atherogenesis^10^, and related endothelial functions^11^. These vascular phenomena are well known to partially regulate perivascular cells^12^; therefore, shear stress-mediated changes in endothelial cell molecular expression may consequently regulate perivascular cell function. Endothelial cell–derived factors, such as endothelin and prostacyclin, reportedly act on perivascular cells, regulating vascular contractility and permeability^13^,^14^. Furthermore, vascular endothelial cell–derived molecules can regulate neuronal network formation during both development and repair processes^15^,^16^. Based on these findings, we hypothesized that endothelial cell–derived molecules, whose production is increased in response to blood flow changes, such as those involved in IPC, may benefit neurons of the central nervous system (CNS) by providing neuroprotective effects against prolonged ischemia.

In this study, we focused on the role of the P2X4 receptor, a subtype of the P2X family of ligand-gated ion channels activated by adenosine triphosphate (ATP). The binding of ATP to the P2X receptor results in the entry of extracellular Ca^2+^ and activates intracellular signaling that evoke a variety of cellular responses. P2X4 is abundantly expressed in vascular endothelial cells^17^,^18^. P2X4^−/−^ mice show weak responses to blood flow changes, such as low Ca^2+^ influx and subsequent NO production^17^, indicating that the P2X4 receptor is a mechanoreceptor that senses blood flow-mediated shear stress. Findings on the role of the P2X4 receptor in vascular pathology have been limited to peripheral vascular diseases, such as heart failure^19^. However, the P2X4 receptor has been detected in the mammalian brain^20^, but its function in the CNS vasculature remains unknown.

Osteopontin, a secreted acidic glycoprotein containing the RGD motif, has multifunctional properties, including adhesive, chemotactic, and cytokine-like properties^21^. Although osteopontin is expressed in the normal brain^21^, the level of osteopontin increases under pathological conditions^22^,^23^. Therefore, the function of osteopontin has been studied in the context of disease. In terms of ischemic brain injury, osteopontin-deficient mice reportedly show increased neurodegeneration after ischemic cortical stroke^24^. Moreover, osteopontin administration prevents infarct formation after stroke in mice^25^. These findings led us to hypothesize that osteopontin is also involved in the endogenous neuroprotective effect mediated by IPC against ischemia, but evidence on this involvement is lacking.

Here, we show that the P2X4 receptor, which is known as a shear stress sensor^17^, is involved in IPC-mediated neuroprotection following middle cerebral artery occlusion (MCAO)-induced cerebral ischemia in mice. P2X4 receptors are expressed by endothelial cells in the adult mouse brain. Treatment with a P2X4 receptor antagonist abolished the ability of IPC to prevent infarct formation and neurological impairment after prolonged ischemia. Shear stress also stimulated P2X4 receptors on vascular endothelial cells, leading to the upregulated expression of osteopontin and a neuroprotective effect^25^. Thus, neuroprotection by P2X receptor-mediated IPC might involve osteopontin up-regulation.

## Results

### P2X4 receptor inhibition abolishes IPC–mediated neuroprotection

**Figure 1:**
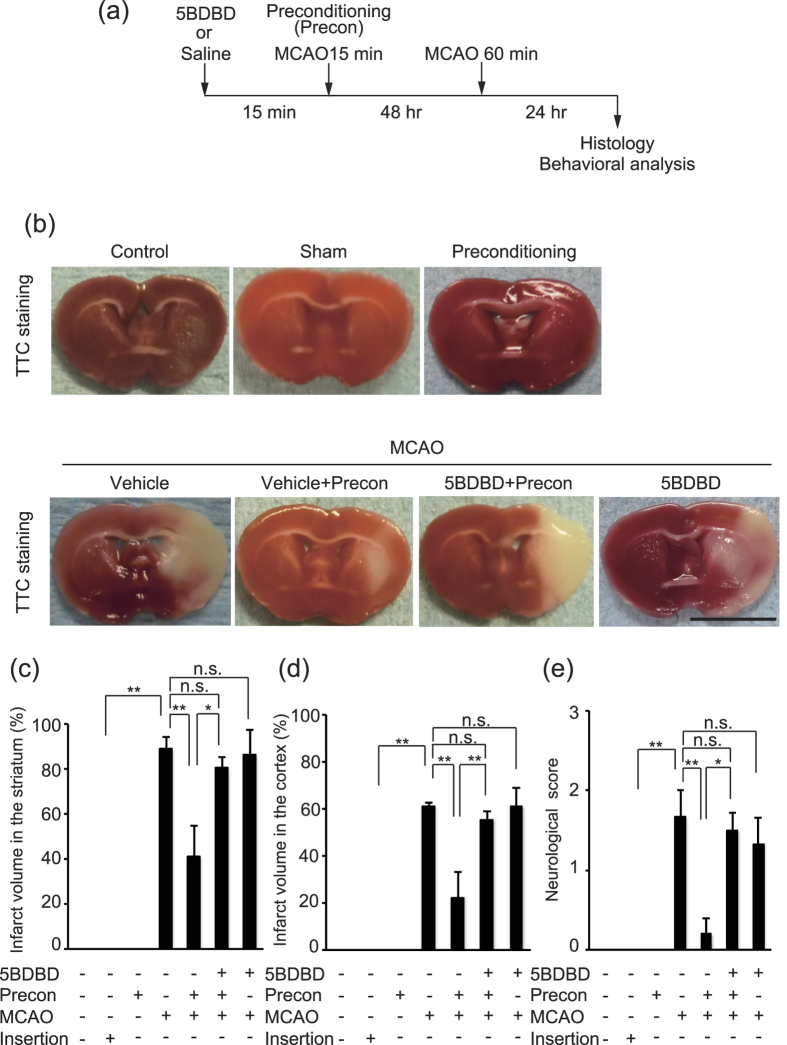
P2X4 receptor inhibition abolishes neuroprotection via ischemic preconditioning. **(a)** Experimental design of the study. IPC (15 min MCAO) was conducted 48 hr before prolonged MCAO (60 min ischemia). 5BDBD or saline was intracerebroventricularly administered 15 min before IPC. Brain sections were obtained 24 hr after prolonged MCAO. **(b)** Representative images of TTC-stained brain slices. Bar, 5 mm. **(c**,**d)** Graphs show the percentage of infarction (infarct ratio) in the ipsilateral striatum (**c**) and cortex (**d**). Insertion means just insert filament into the internal carotid artery which was immediately withdrawn to make sham mice. IPC provided effective neuroprotection against brain injury caused by prolonged MCAO (striatum p = 0.0022, cortex p = 0.0008, **p < 0.01, ANOVA with the Tukey-Kramer test). The infarct volume was larger in mice that underwent both an intracerebroventricular injection of 5-BDBD (148.6 μg/kg body weight), a P2X4 receptor antagonist, and IPC than in mice that did not receive 5BDBD treatment (striatum p = 0.0135, cortex p = 0.0040, *p < 0.05, **p < 0.01, ANOVA with the Tukey-Kramer test). The data represent the mean ± s.e.m. (control, n = 3; IPC, n = 3; MCAO, n = 6; IPC and MCAO, n = 5; 5BDBD, IPC and MCAO, n = 6; 5BDBD and MCAO, n = 3). **(e)** IPC prevented the progression of neurological deficits caused by MCAO (p = 0.0039, **p < 0.01, ANOVA with the Tukey-Kramer test). Mice that underwent the combination of 5-BDBD pretreatment and IPC showed severe neurological deficits compared with those that did not receive 5BDBD treatment (p = 0.0117, *p < 0.05, ANOVA with the Tukey-Kramer test). The data represent the mean ± s.e.m. (control, n = 3; IPC, n = 3; MCAO, n = 6; IPC and MCAO, n = 5; 5BDBD, IPC and MCAO, n = 6; 5BDBD and MCAO, n = 3).

We first established the experimental design (Fig. 1a) and found that the damage obtained 24 hr after 1 hr of MCAO was prevented when 15 min of MCAO (IPC) was administered 2 day before the prolonged MCAO procedure. We then examined the involvement of the P2X4 receptor in the prevention of infarct formation by IPC before prolonged MCAO. Twenty-four hours after 1 hr of MCAO, mice that received an intracerebroventricular injection of 5-BDBD, a P2X4 receptor antagonist, showed larger infarctions in both the striatum and the cortex than controls (Fig. 1b–d). IPC prevents the progression of neurological deficits caused by MCAO, and we consequently also assessed the involvement of the P2X4 receptor in this process. Mice that received a combination of 5-BDBD pretreatment and IPC showed severe neurological deficits compared with those that did not receive 5BDBD treatment (Fig. 1e). We observed that single-IPC did not form an infarct in the brain (Fig. 1b–d) or cause neurological impairment (Fig. 1e). In addition, 5-BDBD treatment did not exacerbate the infarct formation (Fig. 1b–d) or neurological impairment (Fig. 1e) caused by MCAO. Staining with 2,3,5-triphenyltetrazolium chloride (TTC) remained abundant in the brain and no neurological impairment in the intact mice (Fig. 1b–e). These data indicate that P2X4 receptor inhibition abolished IPC–mediated neuroprotection and the prevention of neurological impairment after MCAO.

### P2X4 receptor in vascular endothelial cells is required for neuroprotection against prolonged MCAO

**Figure 2:**
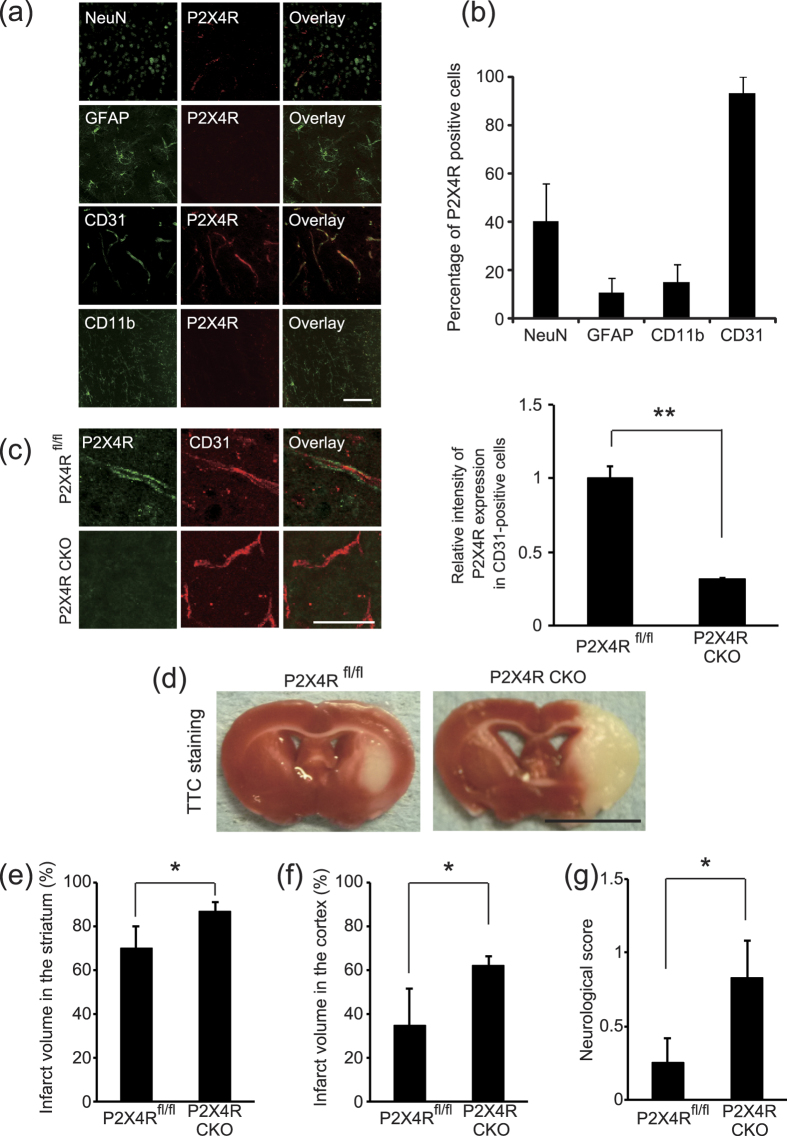
P2X4 receptor expression in vascular endothelial cells is required for neuroprotection after MCAO. **(a)** Immunostaining for P2X4 receptor expression (Alexa Fluor 568) in combination with NeuN, GFAP, CD31, and CD11b (Alexa Fluor 488) in brain sections from intact mice. Scale bar, 100 μm. **(b)** Quantification of P2X4 receptor expression in indicated cell marker-labeled cells. The data represent the mean ± s.e.m. (n = 3). **(c)** Relative intensity of P2X4 receptor protein in CD31-positive cells obtained from the indicated mice treated with tamoxifen (p = 0.0006, **p < 0.01, Student’s *t*-test). Scale bar, 50 μm. Graph shows the intensity of P2X4 receptor expression in CD31-positive cells. The data represent the mean ± s.e.m. (n = 3). (**d**) Representative images of TTC-stained brain slices obtained 24 hr after MCAO. P2X4R fl/fl mice or P2X4R conditional knockout mice (CKO) were subjected IPC 48 hr before MCAO. Bar, 5 mm. (**e,f**) Graphs show the percentage of infarction (infarct ratio) in the ipsilateral striatum (**e**) and cortex (**f**). Genetic ablation of P2X4 receptor in VE-cadherin-positive cells prevented IPC-mediated neuroprotection after prolonged MCAO (striatum p = 0.0497, cortex p = 0.0442, *p < 0.05, ANOVA with the Tukey-Kramer test). The data represent the mean ± s.e.m. (+/+:: flox/flox, n = 4; cre/+:: flox/flox, n = 6). **(g)** P2X4 receptor expression in VE-cadherin-positive cells decreased IPC-prevented neurological deficits caused by MCAO (p = 0.0383, *p < 0.05, ANOVA with the Tukey-Kramer test). The data represent the mean ± s.e.m. (+/+:: flox/flox, n = 4; cre/+:: flox/flox, n = 6).

To investigate the cell type(s) that are involved in P2X4 receptor-mediated neuroprotection, we examined P2X4 receptor expression in the adult mouse brain. Immunohistochemical analysis revealed that CD31-positive vascular endothelial cells expressed P2X4 receptors in the adult mouse brain. In contrast, P2X4 receptors were expressed at very low levels in NeuN-positive neurons, glial fibrillary acidic protein (GFAP)-positive astrocytes, and CD11b-positive microglia (Fig. 2a). Further quantitative analysis revealed that 93.3 ± 6.7% of CD31-positive cells expressed the P2X4 receptor. In other types of cells, P2X4 receptor expression was detected in 40.3 ± 15.7% of NeuN-positive cells, 10.8 ± 5.8% of GFAP-positive cells, and 15 ± 7.6% of CD11b-positive cells (Fig. 2b).

To examine the necessity of P2X4 receptor expression in vascular endothelial cells for IPC-mediated neuroprotection, we generated a conditional knockout mouse in which the P2X4 receptor was knocked down in VE-cadherin-positive vascular endothelial cells. Immunohistochemical analysis confirmed that a decrease in tamoxifen-inducible Cre-mediated recombination decreased P2X4 receptor protein expression in the CD31-positive brain cells of VE-cadherin-Cre/−:: P2X4 receptor flox/flox mice compared with control mice (−/−:: P2X4 receptor flox/flox mice) (Fig. 2c). We then conducted IPC in conditional knockout mice and subjected them to prolonged MCAO. Histological analysis revealed that the infarct area was smaller in the brains of conditional knockout mice than in control mice after MCAO in the striatum and cortex (Fig. 2d–f). In addition, VE-cadherin-Cre/−:: P2X4 receptor flox/flox mice showed severe neurological deficits compared with control mice (−/−:: P2X4 receptor flox/flox mice) (Fig. 2g). These data indicate that the P2X4 receptor is required for IPC-mediated neuroprotection and the prevention of neurological damage after MCAO.

### The P2X4 receptor mediates osteopontin expression in vascular endothelial cells

**Figure 3:**
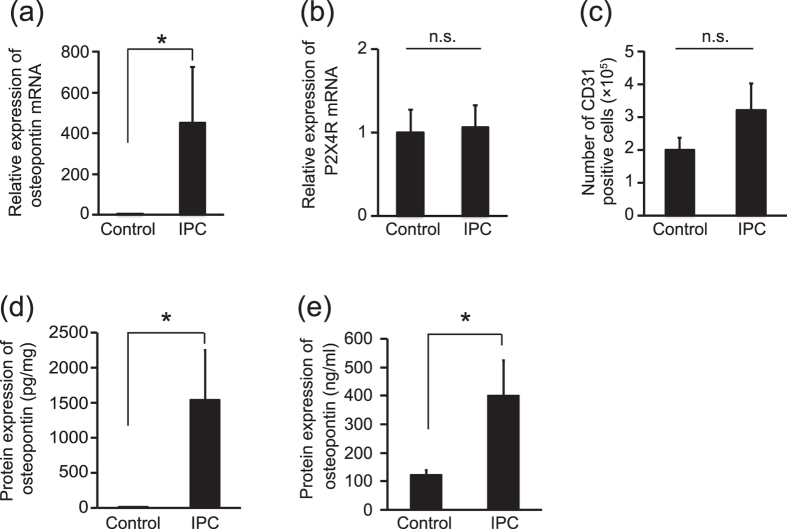
Ischemic preconditioning induces osteopontin expression in P2X4 receptor–expressing vascular endothelial cells. **(a)** IPC upregulated the mRNA expression of osteopontin in CD31-positive cells isolated from mice (p = 0.0376, *p < 0.05, Student’s *t*-test). The data represent the mean ± s.e.m. (IPC, n = 5; Control, n = 7). **(b)** mRNA expression of the P2X4 receptor in CD31-positive cells isolated from mice was not affected by IPC (p = 0.8894, Student’s *t*-test). The data represent the mean ± s.e.m. (IPC, n = 5; Control, n = 7). **(c)** IPC did not change the number of CD31-positive cells in the brain after IPC (p = 0.2108, Student’s *t*-test). The data represent the mean ± s.e.m. (n = 5 each). **(d)** IPC upregulated the expression of osteopontin protein in the ipsilateral hemisphere in mice (p = 0.0304, Student’s *t*-test). The data represent the mean ± s.e.m. (n = 5 each). **(e)** IPC upregulated the expression of osteopontin protein in the peripheral plasma of mice (p = 0.0275, *p < 0.05, Student’s *t*-test). The data represent the mean ± s.e.m. (n = 5 each).

We next investigated the mechanism whereby P2X4 receptors expressed by vascular endothelial cells exert neuroprotective effects via IPC. Vascular endothelial cells are known to secrete factors that support cell survival^26^. Therefore, we hypothesized that IPC would stimulate P2X4 receptor–expressing vascular endothelial cells to enhance the expression of neuroprotective molecules. To test this hypothesis, we screened CD31-positive vascular endothelial cells in the brain for the expression of neuroprotective molecules after IPC and found that IPC increased osteopontin mRNA expression in CD31-positive cells (Fig. 3a) without changing P2X4 receptor expression (Fig. 3b). The number of CD31-positive cells in the brain did not significantly change after IPC (Fig. 3c). These data indicate that IPC induces osteopontin expression in brain vascular endothelial cells that abundantly express P2X4 receptors. We also confirmed that IPC increased the osteopontin protein levels in ischemic hemispheres and peripheral serum (Fig. 3d,e), indicating that the IPC–induced increase in osteopontin may not be a local phenomenon but instead occurs widely throughout the brain.

### Shear stress induces osteopontin expression in vascular endothelial cells via P2X4 receptor activation

**Figure 4:**
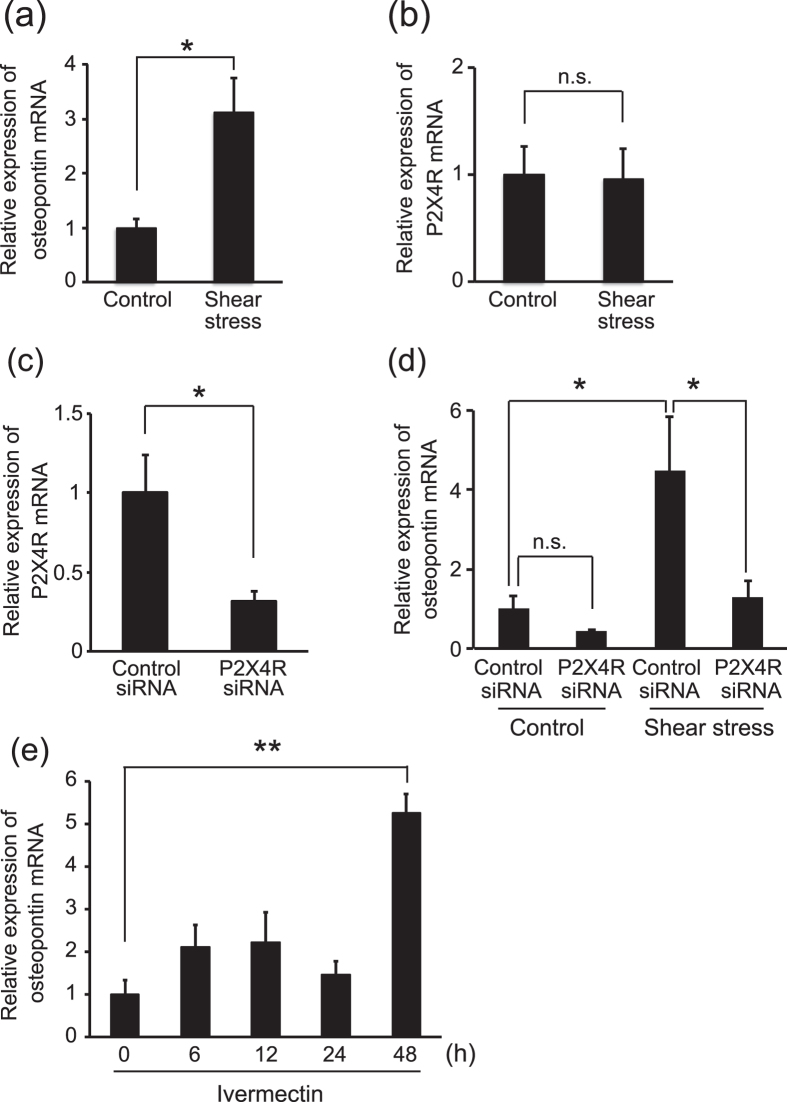
Shear stress induces osteopontin expression in vascular endothelial cells via P2X4 receptor signaling. **(a)** Fluid stimulation (48 hr, 15 dyne/cm^2^ shear stress) upregulated the mRNA expression of osteopontin in b.End3 cells (p = 0.0314, *p < 0.05, Student’s *t*-test). The data represent the mean ± s.e.m. (n = 3 each). **(b)** mRNA expression of the P2X4 receptor in b.End3 cells was not affected by fluid stimulation (48 hr, 15 dyne/cm^2^ shear stress, p = 0.9237 Student’s *t*-test). The data represent the mean ± s.e.m. (n = 6 each). **(c)** mRNA expression of the P2X4 receptor in b.End3 cells was downregulated 72 hr after P2X4 receptor siRNA transfection (p = 0.0341, *p < 0.05, Student’s *t*-test). The data represent the mean ± s.e.m. (n = 4 each). **(d)** Upregulation of osteopontin mRNA in b.End3 cells by fluid stimulation (48 hr, 15 dyne/cm^2^ shear stress) was inhibited by P2X4 receptor siRNA (p = 0.0358, *p < 0.05, ANOVA with Tukey-Kramer test). Cells had previously been transfected with control siRNA or P2X4 receptor siRNA 3 days before fluid stimulation. The data represent the mean ± s.e.m. (Control group, Control siRNA, n = 6; P2X4 receptor siRNA n = 3; Shear stress group, Control siRNA, n = 6; P2X4 receptor siRNA, n = 7). **(e)** mRNA expression of osteopontin in b.End3 cells was upregulated 48 hr after ivermectin treatment (3 μM, p = 0.0003, **p < 0.01, ANOVA with Tukey-Kramer test). The data represent the mean ± s.e.m. (n = 3 each).

P2X4 receptor activation in vascular endothelial cells has been found to induce Ca^2+^ influx into cells^27^ in order to regulate gene expression^28^. In addition, fluid forces reportedly increase osteopontin expression in osteoblast-like MC3T3-E1 cells^29^. Because IPC is defined as a change in blood flow that regulates the shear stress experienced by vascular endothelial cells, we postulated that shear stress stimulates P2X4 receptor activation and consequently promotes osteopontin expression in vascular endothelial cells. To test this postulation, we generated fluid flow to apply shear stress to b.End3 cells, a vascular endothelial cell line derived from the mouse brain. Real-time PCR analysis revealed that shear stress increased the expression of osteopontin mRNA in the culture (Fig. 4a) without significantly affecting P2X4 receptor expression (Fig. 4b). To investigate the involvement of the P2X4 receptor in shear stress-dependent osteopontin expression, we transfected P2X4 receptor siRNA into b.End3 cells (Fig. 4c) and then generated fluid flow within the culture. The silencing of P2X4 receptor expression in b.End3 cells inhibited shear stress-mediated osteopontin expression (Fig. 4d). The P2X4 receptor itself did not affect osteopontin expression (Fig. 4d), suggesting that the latter depends on P2X4 receptor signaling. We also found that treatment with ivermectin, a positive allosteric modulator of the P2X4 receptor, enhanced osteopontin mRNA expression (Fig. 4e). These data confirm that applying shear stress to vascular endothelial cells promotes osteopontin expression via a mechanism that depends on P2X4 receptor signaling.

### Osteopontin administration attenuates increases in infarct formation induced by P2X4 receptor inhibition

**Figure 5:**
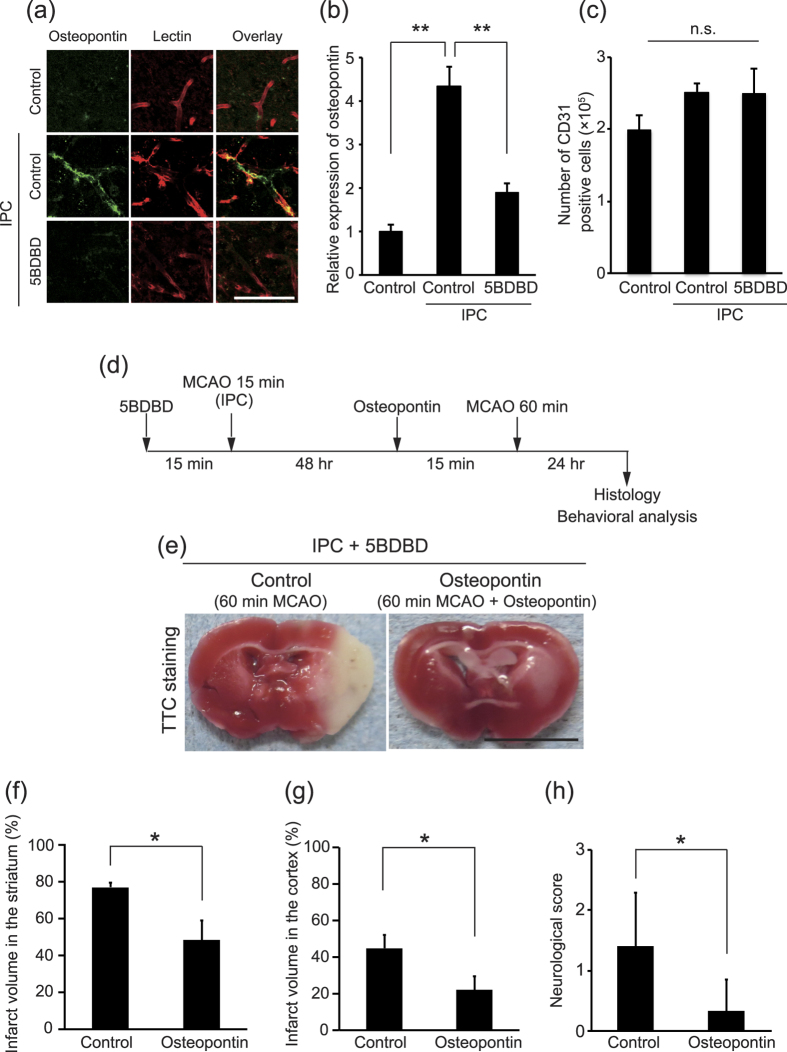
Osteopontin administration attenuates the increase in infarct formation induced by P2X4 receptor inhibition. **(a)** Representative images of brain sections labeled for osteopontin (Alexa Fluor 488) and lectin (Dylight 594). Scale bar, 100 μm. **(b)** IPC upregulated the relative osteopontin expression in lectin-labeled vascular endothelial cells in the brains of mice (p < 0.0001, **p < 0.01, ANOVA with the Tukey-Kramer test). 5BDBD prevented this upregulation (p < 0.0001, **p < 0.01, ANOVA with the Tukey-Kramer test). The data represent the mean ± s.e.m. (n = 3 each). **(c)** The number of CD31-positive cells did not significantly differ between groups (p = 0.2936, ANOVA with the Tukey-Kramer test). The data represent the mean ± s.e.m. (n = 3 each). **(d)** Experimental design of this study. IPC (15 min ischemia) was conducted 48 hr before MCAO (60 min ischemia). 5BDBD was intracerebroventricularly administered 15 min before IPC. Recombinant osteopontin or saline was administered 15 min before MCAO. Brain sections were obtained 24 hr after MCAO. **(e)** Representative images of TTC-stained brain slices. Bar, 5 mm. **(f, g)** Graphs show the percentage of infarction in the ipsilateral striatum **(f)** and in the ipsilateral cortex **(g)**. Osteopontin administration attenuated the increased infarct formation induced by P2X4 receptor inhibition (striatum p = 0.0214, cortex p = 0.0259, *p < 0.05, Student’s *t*-test). The data represent the mean ± s.e.m. (control, n = 5; osteopontin, n = 6). **(h)** Osteopontin administration attenuated the neurological deficit induced by P2X4 receptor inhibition (p = 0.0174, *p < 0.05, Student’s *t*-test). The data represent the mean ± s.e.m. (control, n = 5; osteopontin, n = 6).

We next investigated whether P2X4 receptor-mediated osteopontin expression could be observed in the endothelial cells of mouse brains after IPC. Immunohistochemical analysis revealed a low level of osteopontin expression in CD31-positive endothelial cells in intact mouse brains (Fig. 5a,b). In contrast, CD31-positive cells in mice that had undergone IPC showed an increase in osteopontin expression compared with controls (Fig. 5a,b), suggesting that blood flow changes *in vivo* also promote osteopontin expression in brain endothelial cells. We then addressed whether P2X4 receptor inhibition abrogates IPC-mediated osteopontin expression. We intracerebroventricularly injected mice with 5BDBD and then subjected them to IPC. Mice that underwent 5BDBD treatment before IPC showed reduced osteopontin expression in CD31-positive endothelial cells compared with controls (Fig. 5a,b), and the number of CD31-positive cells in the brain did not significantly differ between groups (Fig. 5c). These data indicate that IPC enhances osteopontin expression in brain vascular endothelial cells through a mechanism that depends on P2X4 receptor activation.

We finally examined whether osteopontin administration is sufficient to exert ischemic tolerance, such as that observed after P2X4 receptor activation. We injected 5BDBD into the cerebral ventricles of mice and then performed transient MCAO, followed by an intracerebroventricular injection of recombinant mouse osteopontin. After prolonged MCAO, we evaluated the infarct volumes and neurological scores of all mice. TTC staining revealed that the infarct volume was smaller in mice treated with osteopontin than in control mice (Fig. 5d–g), indicating that osteopontin treatment mimics the P2X4 receptor-dependent neuroprotective effect of IPC. We also evaluated the neurological deficits in mice treated as described above and found that osteopontin inhibited the exacerbation of neurological deficits after MCAO (Fig. 5h). These results suggest that shear stress induces osteopontin production and prevents neurological dysfunction after brain ischemia.

## Discussion

We found that IPC-mediated neuroprotection in the brain is required for P2X4 receptor signaling. Because the P2X4 antagonist did not affect MCAO-mediated (without IPC) infarct formation, P2X4 receptor inhibition does not prevent MCAO-induced brain damage or neurological deficit. Moreover, the P2X4 receptor has been reported to be a shear stress sensor^17^. Therefore, our findings unveil a novel mechanism of ischemic tolerance, a phenomenon that has previously been considered to be mediated by the regulation of cellular ions and pH homeostasis, mitochondrial modulation, and intracellular energy metabolism^30^. P2 receptors, including the P2X4 receptor, are activated by extracellular adenosine triphosphate (ATP)^31^. Because extracellular ATP concentrations are increased in the striatum during MCAO^32^, extracellular ATP may contribute to the activation of other P2 receptors, which induces ischemic tolerance. In fact, other P2 receptors (P2X7 and P2Y) are known to effectively induce ischemic tolerance^6^,^33^, but this phenomenon has been attributed to changes in neural cells that are the eventual targets of protection after ischemia^34^,^35^,^36^. Regarding P2 expression in vascular endothelial cells, the P2X4 receptor is reportedly the most abundantly expressed P2 receptor in vascular endothelial cells^37^. Thus, we conclude that P2X4 receptor-dependent ischemic tolerance is required for vascular endothelial cells. This idea is supported by the higher level of P2X4 receptor expression observed in vascular endothelial cells, compared with that of astrocytes and microglia, in our study. However, activated microglia in the spinal cord reportedly also express the P2X4 receptor following peripheral nerve injury^38^,^39^, indicating that the expression pattern of the P2X4 receptor may differ by region (brain or spinal cord) and situation (control or injury). To clearly define the role of vascular endothelial cell P2X4 receptors in ischemic tolerance, we generated a conditional knockout mouse in which the P2X4 receptor was knocked down in VE-cadherin-positive vascular endothelial cells. VE-cadherin-Cre/−:: P2X4 receptor flox/flox mice showed large infarction volumes and severe neurological deficits compared with control mice (−/−:: P2X4 receptor flox/flox mice) in response to MCAO after preconditioning. These results indicate that the P2X4 receptors expressed by vascular endothelial cells are related to the acquisition of ischemic tolerance. However, infarct formation between P2X4 receptor knockout and control mice, are significant difference but not so enough. This small difference may be due to the involvement of other cell types (such as neurons or astrocytes)^1^ or another receptor (e.g., P2X7)^6^ in IPC-mediated ischemic tolerance. Additional experiments to investigate the molecular mechanism of IPC-mediated ischemic tolerance are required to understand the contribution of the P2X4 receptor and vascular endothelial cells to ischemic tolerance.

Our *in vitro* experiments revealed the interaction between P2X4 receptor activation and the expression of osteopontin, a well-known neuroprotective factor. P2X4 is a membrane ion channel that elicits high Ca^2+^ influx, even at resting membrane potentials^40^. Furthermore, Ca^2+^ influx stimulates protein kinase C (PKC), which promotes osteopontin expression^41^. Therefore, this intracellular signaling pathway may induce shear stress-mediated osteopontin expression via the P2X4 receptor. We also observed that ivermectin, a positive allosteric modulator of the P2X4 receptor, promoted osteopontin expression in vascular endothelial cells. However, ivermectin, which was developed as an antiparasitic agent, is known to interact with chloride channels in the mammalian CNS and has neurotoxic effects^42^. Thus, candidate therapies that mimic the effects of the P2X4 receptor in terms of inducing ischemic tolerance include a P2X4 receptor activator that lacks toxicity, and osteopontin, a target molecule for P2X4 receptor–mediated neuroprotection.

Osteopontin expression reportedly increases in response to ischemic brain injury^43^,^44^, and microglia and macrophages in the infarct and peri-infarct regions express osteopontin. Although vascular endothelial cells have the potential to synthesize osteopontin^45^, whether vascular endothelial cells express osteopontin after ischemic brain injury has not been clarified. Our study is the first to show that in brain vascular endothelial cells, ischemic injury promotes the expression of osteopontin, which is known to have a neuroprotective effect. Because shear stress regulates the molecular expression of vascular endothelial cells leading to neuroprotection, shear stress may influence neurovascular interaction.

We finally discuss the increase in osteopontin levels in the mouse serum observed in response to IPC in our study. Diffusible molecules produced by organs can enter the circulation and be transported throughout the body; therefore, changes in molecular expression in one organ may influence the function of remote organs. Brief ischemia-reperfusion in remote organs is known to confer myocardial protection, a phenomenon described as remote preconditioning^46^. In this study, we showed that IPC increased the osteopontin levels in both the brain and serum. Based on these findings, we propose that IPC increases the amount of brain osteopontin, which may then leak into the circulation and result in increased serum osteopontin levels. Indeed, osteopontin is known to act as a survival factor in many types of peripheral cells^47^. The scope of our study was limited to the local effects of IPC in the brain; however, future studies focusing on other organs may identify the mechanisms underlying the remote preconditioning effect on other organs and permit the development of methods to treat ischemia throughout the body.

## Methods

### Mice

C57BL/6J mice were obtained from Charles River Japan (Kanagawa, Japan) or Japan SLC (Hamamatsu, Japan). VE cadherin-bacterial artificial chromosome-Cre ERT2 mice were previously described^48^. This study was approved by the institutional committee of Osaka University, and all experiments were performed in accordance with the Guide for the Care and Use of Laboratory Animals of the Graduate School of Medicine of Osaka University (no. 24-067-055).

### Generation of P2X4 receptor floxed mice

The T7 promoter was added to the gRNA template using KOD FX NEO (TOYOBO) and the following primers: P2rx4-1_T7 gRNA_F (5′- TTAATACGACTCACTATAGGagtgttcactgtgcatctccGTTTTAGAGCTAGAAATAGCAAGTTAAAAT -3′), P2rx4-2_T7 gRNA_F (5′-zTTAATACGACTCACTATAGGcctccgggctcccaagagtaGTTTTAGAGCTAGAAATAGCAAGTTAAAAT-3′) and gRNA_common_R2 (5′-AAAAGCACCGACTCGGTGCCACTTTTTCAAGTTGATAACGGACTAGCCTTATTTTAACTTGCTATTTCTAGCTCT-3′). The following Oligo DNA fragments harboring the loxP sequences were obtained from Integrated DNA Technologies: P2X4_loxP1 (5′-GATTGTAGTGTTTAGAGGCTATTTAGACATCCAGTAATAAGAGTAGTGTTCACTGTGCATGGATCCATAACTTCGTATAATGTATGCTATACGAAGTTATCTCCTGGAGCTTGTGACTGTGTTGGCTGACCACAGCGTTCATTTCTCACATAATAACCAG-3′) and P2X4_loxP2 (5′-TAGCTGGCTCCAACCCAACACTCTGTCTTTCCCACACCTTCATGCCTCCGGGCTCCCAAGGGATCCATAACTTCGTATAATGTATGCTATACGAAGTTATAGTAAGGAAACAAAGGTCTTAAAAGTCCTGGGAAGCAAGGCAAGAAATAACACCTTCTGC-3′). The T7- P2rx4-1 or – P2rx4-2 gRNA PCR product was gel purified and used to subsequently generate gRNA. MEGAshortscript T7 (Life Technologies) was used to generate the gRNA. Cas9 mRNA was generated by *in vitro* transcription (IVT) using the mMESSAGE mMACHINE T7 ULTRA kit (Life Technologies), and the template that was amplified by PCR using pEF6-hCas9-Puro and the primers T7Cas9_IVT_F and Cas9_R^49^ was gel-purified.

The synthesized gRNA, Cas9 mRNA and oligo DNA fragments harboring the loxP sequences were purified using the MEGAclear kit (Life Technologies) and eluted in RNase-free water (Nacalai Tesque). To obtain P2rx4-floxed mice, C57BL/6 female mice (6 weeks old) were superovulated and mated to C57BL/6 stud males. Fertilized one-cell-stage embryos were collected from oviducts and injected into the pronuclei or cytoplasm with the Cas9 mRNA (100 ng/μl), the gRNA (50 ng/μl) and the oligo DNA fragments (100 ng/μl), as previously described^50^. The injected live embryos were transferred into the oviducts of pseudopregnant ICR females at 0.5 dpc, as previously described^50^. The pups were screened by PCR using primers P2rx4_loxP1_F (5′-CGGCTGGCTTAACCATTCTTTGTCT-3′) and P2rx4_loxP1_R (5′-agtgttggtcacagccacacctttg-3′) as well as P2rx4_loxP2_F (5′-caaaggtgtggctgtgaccaacact-3′) and P2rx4_loxP2_R (5′-TTAACAGTGTGTAGGTGAGGATGGC-3′) to test whether the loxP sequences had been correctly inserted. Pups exhibiting correct loxP insertion were mated to C57BL/6 female or male mice and tested for germ line transmission.

The vascular endothelial cell-specific P2X4 receptor deletion mice were obtained by crossing the P2X4 receptor flox mice with the VE-cadherin creERT2 mice. Cre recombination in the generated mice was induced by administering tamoxifen (100 mg/kg, i.p.; Sigma-Aldrich) daily over 5 consecutive days. Fourteen days after the first tamoxifen administration, vascular endothelial cells were obtained from the brains of Cre/−:: flox/flox mice and −/−:: flox/flox mice using CD31-specific antibody-coated magnetic beads (Miltenyi-Biotech) to assess the efficiency of P2X4 receptor deletion. The relative P2X4 receptor expression was assessed by immunohistological analysis.

### Surgical procedures

The experimental design is described in Figs 1a and 5d. Adult male mice (8 weeks) were anesthetized via isoflurane inhalation, and MCAO was induced by the intraluminal insertion of a silicon-coated nylon filament (Re L910 PK5, Doccol Corporation) via the proximal external carotid artery into the internal carotid artery. CBF was monitored by laser Doppler flowmetry using a probe located in the ipsilateral parietal bone (1–2 mm posterior to bregma), and a >90% reduction in CSF was considered to indicate successful occlusion. The head temperatures were maintained at 36 °C using a warming lamp. To induce IPC (15 min ischemia), the filament was inserted through the external carotid artery to the internal carotid artery and left in place for 15 min. IPC was conducted 48 hr before MCAO. Sham animals were generated by the insertion of a filament into the internal carotid artery which was immediately withdrawn. To induce transient MCAO, the filament was inserted through the common carotid artery into the internal carotid artery and left in place for 60 min. The P2X4 receptor antagonist 5-(3-bromophenyl)-1, 3-dihydro-*2H*-benzofuro[3, 2-*e*]-1, 4-diazepin-2-one (5-BDBD, 148.6 μg/kg body weight, Tocris Bioscience)^51^,^52^,^53^,^54^ and mouse recombinant osteopontin (0.42 μg/kg, Calbiochem) were slowly injected into the right lateral ventricle (coordinates from bregma: 1 mm lateral, 0 mm posterior, 2 μl per site, at a depth of 1.8 mm from the brain surface) using a glass capillary tube attached to a microsyringe. 5BDBD or saline was intracerebroventricularly administered 15 min before IPC. Recombinant osteopontin or saline was administered 15 min before MCAO. We confirmed that preconditioning (15 min, MCAO) prevented the formation severe brain infarcts (Fig. 1b‒d). We also observed no difference in the head temperature between any of the animal groups during any of the surgical procedures (data not shown).

### Histology and immunohistochemistry

One day after transient MCAO, the mice were transcardially perfused with cold saline. The brains were cut into serial coronal slices of 1 mm thickness, and the slices were stained with 0.4% 2,3,5-triphenyltetrazolium chloride (TTC) for 20 min at 37 °C followed by fixation with 4% paraformaldehyde (PFA). To estimate the infarct area ratio, the TTC-negative area in each slice was determined by subtracting the area of intact tissue in the ischemic hemisphere from the total area of the intact contralateral hemisphere to correct for brain edema^55^. The sum of the infarct area in all slices was multiplied by the slice thickness (1 mm) to provide the infarct volume. The infarct area ratio was calculated as follows: (infarct volume)/(ipsilateral hemisphere volume) ×100.

For immunohistochemistry, the mice were transcardially perfused with 4% PFA in PBS, and the brains were post-fixed with 4% PFA at 4 °C overnight, followed by immersion in 30% sucrose in PBS. The brains were embedded in optimal cutting temperature compound (Tissue-Tek, Sakura Finetek) for frozen sectioning. Coronal sections were cut at a 30-μm thickness on a cryostat and mounted on Matsunami adhesive silane-coated slides (Matsunami Glass). The sections were treated with PBS containing 5% bovine serum albumin (BSA, Sigma-Aldrich) and 0.1% Triton X-100 for 1 hr at room temperature. The sections were then incubated with primary antibodies diluted in PBS containing 5% BSA and 0.1% Triton X-100 overnight at 4 °C and subsequently incubated with secondary antibodies for 1 hr at room temperature. The following antibodies were used: rabbit anti-P2X4 receptor (Millipore), goat anti-osteopontin (R&D Systems), mouse anti-NeuN (Millipore), mouse anti-GFAP (Sigma-Aldrich), rat anti-CD31 (BD Biosciences), rat anti-CD11b (BD PharMingen), AlexaFluor 568 anti-rabbit IgG (Invitrogen), AlexaFluor 488 anti-mouse IgG (Invitrogen), AlexaFluor 488 anti-rat IgG (Invitrogen), and AlexaFluor 488 anti-goat IgG (Invitrogen). To visualize the vasculature, the sections were stained with Dylight 594–labeled lectin (Vector Laboratories). Images were acquired with a confocal laser-scanning microscope (Olympus FluoView FV1200).

To quantitatively analyze osteopontin expression, we randomly selected 30 lectin-labeled vessels in three cerebral cortex sections and measured the fluorescence intensity of osteopontin expression in lectin-positive vessels. The average fluorescence intensity from one mouse comprised one data point, and we calculated the relative intensity based on the mean intensity in the control group.

### Neurological evaluation

Neurological deficits of mice were scored as previously described^56^ using the following scheme: 0, no observable neurological deficits (normal); 1, failure to extend right forepaw (mild); 2, circling to the contralateral side (moderate); and 3, loss of walking or righting reflex (severe).

### CD31-positive cell isolation

Two days after IPC, the mice were transcardially perfused with cold saline. The brains were dissected in PBC and dissociated into single-cell suspensions using a Neural Tissue Dissociation Kit (P) (Miltenyi-Biotec). CD31-positive cells were isolated using CD31-specific antibody-coated magnetic beads (Miltenyi-Biotec).

### Quantitative real-time reverse transcription polymerase chain reaction (qRT-PCR)

RNA was extracted from tissues and cells using an RNeasy Mini Kit (Qiagen) and reverse transcribed using the Prime Script II High Fidelity RT-PCR Kit (Takara) or RT2 First Strand Kit (Qiagen). Quantitative PCR was performed using an ABI ViiA7 real-time PCR system (Applied Biosystems). The PCR was performed with Power SYBR Green PCR Master Mix (Applied Biosystems) at a primer concentration of 100 nM. The PCR conditions consisted of 1 cycle at 95 °C for 10 min, 40 cycles of 95 °C for 15 s, and 60 °C for 60 s. The following primers were used: osteopontin forward AGCCACAAGTTTCACAGCCACAAGG, osteopontin reverse CTGAGAAATGAGCAGTTAGTATTCCTGC, P2X4R forward CCCTTTGCCTGCCCAGATAT, P2X4R reverse CCGTACGCCTTGGTGAGTGT, GAPDH forward TGTGTCCGTCGTGGATCTGA, and GAPDH reverse TTGCTGTTGAAGTCGCAGGAG. Melting curve analysis was carried out following PCR to monitor amplicon specificity. The cycle threshold (Ct) values were calculated using the ΔΔCt method to obtain the fold differences. Each mRNA value was normalized to the level of GAPDH.

To identify candidate molecules that may be responsible for ischemic tolerance, we collected CD31-positive cells from mouse brains after IPC. Changes in the mRNA expression of CD31-positive cells were measured using a Mouse Neurotrophin and Receptors PCR Array (Qiagen).

### Enzyme-linked immunosorbent assay (ELISA)

The osteopontin levels in mouse serum or tissue lysates were examined using a Mouse Osteopontin Quantikine ELISA kit (R&D Systems).

### Cell culture experiments

b.End3 cells were obtained from the American Type Culture Collection. The cells were maintained at 37 °C under 5% CO_2_ and cultured in Dulbecco’s modified Eagle’s medium (DMEM, Sigma-Aldrich) supplemented with 10% (vol/vol) heat-inactivated fetal bovine serum (FBS, Gibco). To induce fluid shear stress (15 dynes/cm^2^)^57^, the cells were cultured in a perfusion chamber (Nepagene) that was connected to a peristaltic pump (AC-2120, ATTO) by silastic tubing.

Mouse P2X4R siRNA and nonsense siRNA (Silencer negative control siRNA) were purchased from by Life Technologies (Stealth siRNAs). The sense and antisense siRNA strands were defined as follows: 5′-GCUUUGACAUCAUCGUGUUUGGAAA-3′ (sense) and 5′-UUUCCAAACACGAUGUCAAAGC-3′ (antisense). P2X4R siRNA was transfected into cultured b.End3 cells using Lipofectamine RNAiMAX reagent (Life Technologies). The cells were lysed 72 hr after transfection and then subjected to real-time PCR analysis.

### Statistical analysis

The data are presented as the mean ± s.e.m. The significance of differences between groups was examined using Student’s *t*-test or one-way analysis of variance (ANOVA) followed by the Tukey-Kramer test. *P* values of less than 0.05 were considered to be significant.

## Additional Information

**How to cite this article**: Ozaki, T. *et al.* The P2X4 receptor is required for neuroprotection via ischemic preconditioning. *Sci. Rep.* **6**, 25893; doi: 10.1038/srep25893 (2016).
